# Resolutions

**Published:** 1901-04

**Authors:** 


### RESOLUTIONS.

*The following Resolutions were passed at the Annual Meeting of the Pennsylvania State Veterinary Medical Association, March* 5 *and* 6, 1901:

### DEATH OF DR. A. W. CLEMENT.

WHEREAS, The Pennsylvania State Veterinary Medical Association, in annual convention assembled in the city of Philadelphia, has received the sad intelligence of the death of Dr. A. W. Clement, of Baltimore, Md., former President of the American Veterinary Medical Association; and

WHEREAS, Dr. Clement throughout his professional career always stood for higher veterinary education and the advancement of our science and art in general, rendering distinguished services for the profession in his own State and the nation; therefore be it

*Resolved*, That the Pennsylvania State Veterinary Medical Association extend to his family their heartfelt sympathy in this their hour of bereavement, and to the profession of his State and the nation their deep regret and loss in his untimely death.

### DEATH OF DR. N. E. REINHART.

WHEREAS, It has pleased Almighty God to remove from our midst our late member, Dr. N. E. Reinhart; and

WHEREAS, His activity as a member of this Association, as an ardent devotee of the profession, as a firm friend and good citizen make his loss a marked one, and while his death was a relief from great physical suffering and distress it has brought to us the loss of a faithful associate; therefore be it

*Resolved*, That we extend to his family our deep sympathy in their affliction, and record our appreciation of his energy, faithfulness, and devotion. Be it further

*Resolved*, That a copy of these resolutions be spread upon the minutes and that a copy be sent to the bereaved family.

### A. V. M. A.

WHEREAS, Through the efforts of the veterinarians in the East, and especially the members of the Veterinary Medical Association of New Jersey and the veterinary journals, the American Veterinary Medical Association is to hold its meeting for 1901 at Atlantic City, September 3d, 4th, and 5th; and

WHEREAS, The attractions of this famous seaside resort, its facilities for caring for and entertaining all who may attend, and its accessibility to all parts of the field of the activity of the early years of the Association make it especially favorable for this purpose; and

WHEREAS, The veterinarians of New Jersey are now all united in one strong organization, working as a unit for the good of the profession; be it

*Resolved*, That we regard it as a duty of our members to assist in every way possible to make this meeting a success, and we urge them to attend and to use every effort to make this the best meeting of its kind that has ever been held and a fitting opening of the National Association work of the new century.

### ONE CLAUDE D. MORRIS.

WHEREAS, The defeat of our proposed Army Veterinary Corps was contributed to by one Claude D. Morris, acting ostensibly as the Secretary of the New York State Veterinary Medical Association; and

WHEREAS, In so doing he deliberately traduced the practically unanimous opinion of that organization, maligned the good name of the profession in the Empire State and in the country, and was utterly forgetful of personal honor, duty, and integrity; and

WHEREAS, Such actions by any man within professional circles, associations or organizations should receive the severe condemnation of every member of the profession who has its honor at heart; therefore, be it

*Resolved,* That we, the members of the Pennsylvania State Veterinary Medical Association, in convention assembled, brand such actions as those of a Judas Iscariot and call upon our colleagues in New York State to summarily expel him from their organization with the brand of a traitor on his forehead.

### RABIES.

WHEREAS, It appears that rabies is becoming more prevalent and that there is not at this time any adequate provision for the execution of the measures that it is necessary to take to keep this dangerous disease in check; be it

*Resolved,* That this Association favors the passage of an act that will enable the State Live-stock Sanitary Board to adopt and enforce such quarantine and other measures as will serve to prevent the spread of rabies;

WHEREAS, It is proposed to enact a law whereby animals bitten by a rabid dog may be paid for in the manner now followed in paying for sheep injured or killed by dogs; and

WHEREAS, It appears to be eminently just and proper to pay for other animals injured by dogs as well as sheep; be it

*Resolved,* That this Association cordially approves House Bill No. 91, introduced by Hon. Jeremiah Roth, with the exception that the price of horses and mules compensated for should be limited to $40 per head and of cattle to $25 per head if unregistered, and $50 per head if registered.

### PROPOSED VETERINARY SCHOOL.

WHEREAS, We have heard of the proposed institution of another veterinary school in the city of Philadelphia; and

WHEREAS, We believe that the existing veterinary school of the University of Pennsylvania fully covers the field and offers ample facilities for all seeking education as veterinarians; therefore be it

*Resolved,* That this Association express its disapproval of the formation of any additional school

### DR. LEONARD PEARSON.

WHEREAS, The profession of our State has been blessed by many able leaders in all movements for general advancement of the profession; and

WHEREAS, Its progress in the last ten years has been contributed to in greater measure by one of our number in educational work, in strengthening fraternal relations, in broader and better sanitary control measures, in association advancement, and in all directions conducive to the higher attainments of our profession; therefore be it

*Resolved,* That in this mark of recognition tendered our able leader, our State Veterinarian, the Dean of our State Veterinary College, the friend of every true veterinarian in our State and country, we extend our sincere appreciation of such a valued member and guide, and desire to place on the records of our Association our heartfelt thanks to Dr. Leonard Pearson.

